# A Novel Integrated Strategy for Discovering Absorbable Anticoagulant Bioactive Peptides: A Case Study on Leech Protein Hydrolysates

**DOI:** 10.3390/molecules30153184

**Published:** 2025-07-30

**Authors:** Ke-Xin Fang, Xi Sun, Liang-Ke Chen, Kun Wang, Chao-Jie Yang, Shan-Shan Mei, Chu-Ying Huang, Yao-Jun Yang

**Affiliations:** School of Chinese Materia Medica, Beijing University of Chinese Medicine, Beijing 102488, China; fkxflos@163.com (K.-X.F.); sunxi146@163.com (X.S.); 18811386098@163.com (L.-K.C.); 15176041828@163.com (K.W.); 20230941459@bucm.edu.cn (C.-J.Y.); 20230935208@bucm.edu.cn (S.-S.M.); 20230935207@bucm.edu.cn (C.-Y.H.)

**Keywords:** leech (*Whitmania pigra* Whitman), bioactive components, anticoagulant activity, Critic-G1 method, peptidomics

## Abstract

Medicinal plants and animal-derived proteins represent valuable natural sources of bioactive components with pharmaceutical potential. Whilst some medicinal plants and animal-derived proteins also offer rich sources of anticoagulant bioactive peptides, their development faces multiple challenges: anticoagulant evaluation relies on single-parameter assays with limited reliability, native proteins demonstrate suboptimal activity without enzymatic treatment, and few researchers investigate bioavailable peptides. Our study establishes an innovative framework using the leech as a case study to overcome these barriers. A novel anticoagulant evaluation model was first established with the Critic-G1 weighting method. And we optimized the enzymatically hydrolyzed extracts with high activity using Box–Behnken response surface methodology. Subsequently, the everted gut sac model was implemented to simulate intestinal absorption and screen for absorbable peptide fractions. Furthermore, peptidomics was employed to identify the bioactive peptides. Lastly, we identified the bioactivity using anticoagulation assays. Results indicated that the optimal hydrolysis conditions were achieved with trypsin at 50.48 °C, an enzyme-to-substrate ratio of 6.78%, 7.51 h, and pH of 8.06. The peptide DLRWM was identified through integrated peptidomics and molecular docking approaches, with subsequent activity validation demonstrating its potent anticoagulant effects. This study has successfully identified a novel anticoagulant peptide (DLRWM) with confirmed intestinal absorption properties and provides a template for unlocking the pharmaceutical potential of medicinal animal proteins.

## 1. Introduction

Coagulation disorders include various blood-related conditions. These conditions manifest as either excessive bleeding or abnormal clotting. They pose serious challenges for diagnosis and treatment in clinical settings worldwide. Accurate evaluation and treatment rely on standard coagulation tests. The main tests are activated partial thromboplastin time (APTT), prothrombin time (PT), and thrombin time (TT). Each test offers unique information about different stages of the complex coagulation process. Currently, APTT, PT, and TT assays are the most commonly employed methods for assessing coagulation function [1]. However, when evaluating the anticoagulant activity each coagulation parameter must be independently measured and analyzed [2]. This not only increases the complexity and workload of the assessment process but also presents analytical challenges [3]. Furthermore, due to the inherent variability among individual coagulation indicators, the resulting conclusions may lack consistency and reliability. Therefore, we aimed to develop a mathematical evaluation method integrating multiple parameters. By harmonizing subjective expert judgment (G1 method) with objective data-driven analysis (Critic method), this approach enables a more accurate and multidimensional evaluation of anticoagulant efficacy [4]. Such integrative models have been successfully applied in optimizing processing techniques [5].

*Whitmania pigra* Whitman (WP), a species of leech widely used in Traditional Chinese Medicine (TCM), has been renowned for its remarkable blood-activating and stasis-resolving properties [6]. As a key TCM, it is clinically applied to treat conditions such as blood stasis-induced amenorrhea, cerebrovascular diseases, and trauma-related injuries [7]. Modern pharmacological studies have revealed that WP exhibits potent anticoagulant, antithrombotic, and antiplatelet aggregation activities, primarily attributed to its proteinaceous, peptide components and their derived peptides [8]. Unlike hematophagous leeches (e.g., *Hirudo nipponica*), which produce the well-known anticoagulant hirudin, WP does not feed on blood but preys on mollusks [9]. Despite the absence of hirudin, its anticoagulant activity persists, suggesting the presence of unique bioactive peptides yet to be fully characterized [10]. This specific property of WP makes it highly promising for developing anticoagulants.

Protein-based natural medicines, derived from either plant or animal sources, typically require enzymatic digestion following oral administration to release bioactive peptides that exert physiological effects in the body [11]. Compared with traditional extraction methods such as solvent extraction or mechanical disruption, enzymatic hydrolysis has been shown to significantly enhance the release and activity of bioactive peptides [12]. Owing to its mild reaction conditions and high specificity, enzymatic hydrolysis has emerged as a preferred strategy in the development of active components from natural sources [13]. For protein-rich natural medicines, optimizing key enzymatic parameters can further improve peptide yield and functional specificity, thereby facilitating bioactivity screening and pharmacological investigation [14]. Response surface methodology (RSM), particularly the Box–Behnken design (BBD), provides a robust platform to optimize enzymatic parameters (e.g., protease type, temperature, pH) whilst accounting for interactions between variables. By employing RSM we can systematically screen for optimal enzymatic conditions to obtain hydrolysates with maximum anticoagulant activity, thereby increasing the likelihood of identifying highly effective anticoagulant peptides [15]. However, achieving high in vitro activity alone is not sufficient; the clinical efficacy of peptide-based agents also depends on their ability to be absorbed and remain stable in the gastrointestinal environment. Despite progress in screening bioactive peptides, relatively few studies have addressed whether such peptides can cross the intestinal barrier and exert effects in vivo [16]. To bridge this gap the everted gut sac model, recognized as a rapid in vitro screening tool, serves as a reliable method for evaluating intestinal absorption potential and metabolic stability [17]. Mass spectrometry-based peptidomics methods are widely applied for the identification of unknown peptides [18]. This method has now been widely used for the identification of bioactive peptides. Molecular docking simulations further enable the prediction of peptide-target interactions, offering insights into potential anticoagulant peptides by targeting thrombin or other coagulation factors [19].

Herein, in this study we propose an integrated strategy aimed at addressing current gaps in the evaluation and screening of bioactive peptides from WP. To achieve this, we first established a Critic-G1-based anticoagulant evaluation model that integrates multiple parameters, including APTT, PT, TT, and peptide content. Using this model as a benchmark, we employed response surface methodology (RSM) to optimize the hydrolysis conditions of WP, thereby obtaining hydrolysates with the highest anticoagulant efficacy. Subsequently, the everted intestinal sac model was utilized to screen for peptide fractions with potential absorption. These bioactive peptides were then identified through Nano-LC-MS/MS analysis combined with molecular docking studies. Finally, the anticoagulant activities of synthetic peptides were validated via in vitro assays. This study represents the application of an integrated mathematical modeling–experimental approach for screening bioactive peptides of WP, significantly advancing the understanding of WP’s bioactive components and providing a template for studying protein-based natural products.

## 2. Results

### 2.1. Determination of Anticoagulant Evaluation Indicators

The four coagulation tests are commonly used in clinical practice to diagnose coagulation dysfunction or thrombotic diseases [20]. APTT mainly reflects the status of the intrinsic coagulation system, PT mainly reflects the status of the extrinsic coagulation system, TT mainly reflects the time required for fibrinogen to convert into fibrin, and FIB mainly reflects the content of fibrinogen. However, FIB testing has high demands on the instruments and testing conditions, making it unsuitable as a standard for detection. Therefore, this study selected three testing indicators—APTT, PT, and TT—along with peptide content, to collectively reflect the anticoagulant function of the WP enzyme hydrolysate. As shown in Table 1, the WP enzyme hydrolysate peptides significantly prolonged APTT, PT, and TT. Specifically, the APTT prolongation rate ranged from 29% to 36%, the PT prolongation rate was between 16% and 19%, and the TT prolongation rate reached 102% to 120%. We found that the WP enzyme hydrolysate peptides can simultaneously affect both the intrinsic and extrinsic coagulation pathways.

### 2.2. Comprehensive Evaluation Indicators Established by Critic-G1

The anticoagulant evaluation metrics and peptide values were assigned weights using three weighting methods (Critic, G1, and Critic-G1) to obtain W1, W2, and W. The results are shown in Table 2. Intuitive analysis shows that the comprehensive score results obtained from the three methods do not differ significantly.

A comprehensive score was generated through the application of weighting coefficients derived from the following three independent weighting methodologies: Critic, G1, and their combination Critic-G1. The results are shown in Table 3. The data were analyzed using SPSS 22.0. Correlation coefficient analysis shows that the correlation coefficient between Critic and Critic-G1 hybrid weighting methods is 0.990, between the G1 and Critic-G1 methods is 0.872, and between the Critic and G1 methods is 0.816. The correlations among the three methods are statistically significant (*p* < 0.01), indicating that the comprehensive scores from the three methods are consistent. As for the weight coefficients, the correlation coefficient between Critic and G1 is 0.197, with no statistical significance (*p* > 0.05), suggesting that the information reflected by the two methods is not additive.

Upon comparison it is found that the Critic-G1 hybrid weighting method takes both subjective and objective factors into account, resulting in more scientific and reasonable information, and the obtained weights (Ws) have higher reliability. Therefore, this study ultimately selects the weight coefficients determined by the Critic-G1 hybrid weighting method to calculate the comprehensive scores of each indicator, which is shown as follows:

(1) $$Z=\left(\frac{APTT}{APTTmax}\times 0.1254+\frac{PT}{PTmax}\times 0.2544+\frac{TT}{TTmax}\times 0.3726+\frac{C}{Cmax}\times 0.2475\right)$$

where Z represents comprehensive score and C represents WP enzyme hydrolysis peptide content. This study is the first to combine anticoagulation indicators and peptide content, establishing a weighted equation for comprehensive evaluation. This evaluation method is also used as the response value in the BBD analysis discussed later.

### 2.3. BBD-RSM Model Fitting and Analysis

**Figure 1:**
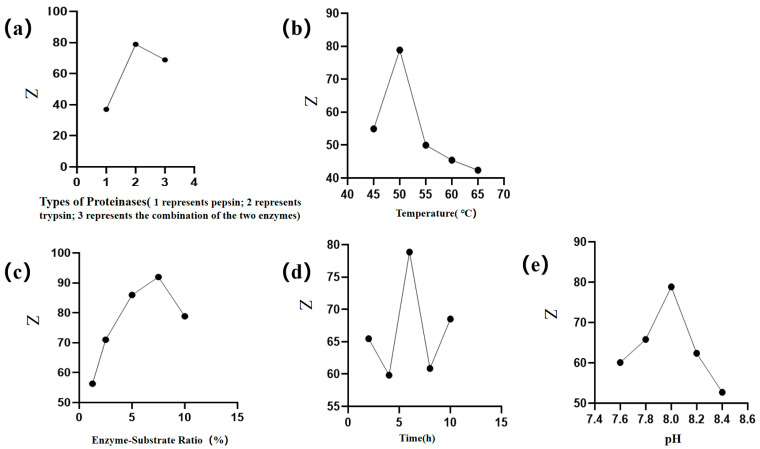
Single-factor experimental results of WP enzymatic hydrolysis process. Dependence of comprehensive score on reaction parameters (**a**) protease type, (**b**) temperature, (**c**) enzyme-to-substrate ratio, (**d**) time, and (**e**) pH.

The results shown in Figure 1 reflect the impact of different factors on the anticoagulation evaluation indicators and peptide content. Both contents increased initially, followed by a subsequent decrease. As single-factor experiments consider only one variable at a time and do not account for interactions between factors, they may lead to locally optimal conditions that are not necessarily suitable when multiple variables interact. Therefore, BBD was employed to further explore the joint effects of key factors and identify a more robust and globally optimized combination [21]. For the BBD we selected the point with the highest response value and its immediate neighbors to establish the following three distinct values, as detailed below: temperature at 45 °C, 50 °C, and 55 °C; enzyme-to-substrate ratio (5%, 7.5%, 10%); and hydrolysis time (4 h, 6 h, 8 h), pH (7.8, 8.0, 8.2).

Comprehensive score (Z) was set as the dependent variable, with factors A, B, C, and D as independent variables. A represents the enzymatic hydrolysis time, B represents the enzyme–substrate ratio, C represents the enzymatic hydrolysis temperature, and D represents the pH value. Using Design-Expert 8.0.6 software, a second-order multiple regression model analysis was conducted, yielding the regression equation, as shown in the following Equation (2):

(2) $$Z=69.80-4.90*A+3.55*B+4.09*C+2.28*D+1.36*AB+10.84*AC\;\;-3.94*AD-5.36*BC-16.04*BD+4.82*CD-7.74*A^{2}\;-12.23*B^{2}-3.58*C^{2}-12.89*D^{2}$$

The results of the analysis of variance are shown in Supplementary Material Table S1. The variance analysis was performed on the multivariate quadratic regression model Y (Supplementary Material Table S2). The F-value of the quadratic regression model was 37.47 (*p* < 0.0001), indicating that the model is statistically significant and suitable for analysis and prediction. The R^2^ of the fitted model was 0.9740, with R^2^_Adj_ and R^2^_Pred_ being 0.9480 and 0.8756, respectively, indicating that the model explains a high proportion of the variation in the process. A CV% of 5.23% (<10%) demonstrates good precision of the experimental data. The Adeq Precision value of 22.964 (>4) is generally considered desirable. These results collectively confirm that the fitted regression equation satisfies all validation criteria, demonstrates good predictive capability, and can be effectively used to analyze and predict the enzymatic hydrolysis process for WP.

**Figure 2:**
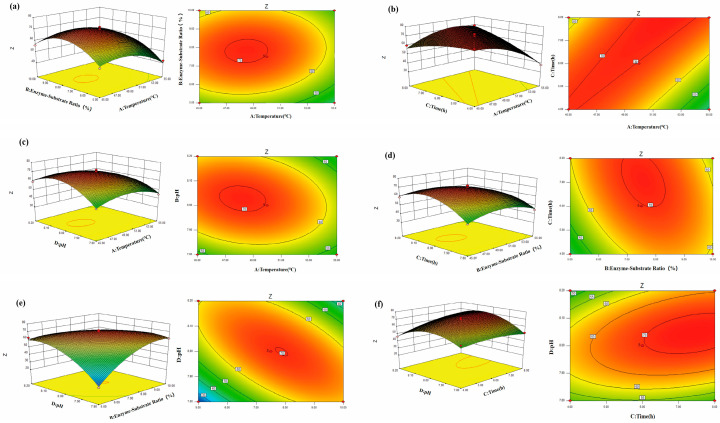
The effect of different influencing factors on the comprehensive anticoagulation score of WP. (**a**) Temperature and enzyme–substrate ratio; (**b**) temperature and time; (**c**) temperature and pH; (**d**) enzyme–substrate ratio and time; (**e**) enzyme–substrate ratio and pH; (**f**) time and pH.

To further investigate the interaction effects of the factors, this study used Design-Expert 8.0.6 software to generate response surface and contour plots. The response surface and contour plots reflect the influence of the interaction between two factors from three-dimensional and two-dimensional perspectives. As shown in Figure 2, the contour lines of the comprehensive anticoagulation score for WP are nearly elliptical, indicating that the interaction between the enzyme–substrate ratio and enzymatic hydrolysis time, as well as between enzymatic hydrolysis time and pH value, is significant, consistent with the model results. The steeper the response surface, the greater the influence on the comprehensive anticoagulation score of WP. Therefore, the plots visually reflect the influence of the interaction between various factors on the comprehensive anticoagulation score, which is consistent with the results of the variance analysis.

Based on the condition of maximizing the comprehensive score for the WP enzymatic hydrolysis process, the following optimal enzymatic hydrolysis conditions were obtained through fitting with Design-Expert 8.0.6 software: trypsin as the enzyme, the temperature was 50.48 °C, the enzyme–substrate ratio was 6.78%, the hydrolysis time was 7.51 h, and the pH value of the hydrolysis was 8.06. The comprehensive score at this point was 72.07. After obtaining the predicted values, we conducted a validation. Three parallel validation experiments were conducted using the optimal enzymatic hydrolysis conditions, yielding comprehensive scores of 71.62, 72.10, and 72.02. The average comprehensive score was 71.91 (RSD = 0.36%, *n* = 3), with a relative error of 0.22% compared to the predicted value of 72.07, indicating that the accuracy and stability of the WP enzymatic hydrolysis process are both good.

### 2.4. MS Identification and in Silico Screening

**Figure 3:**
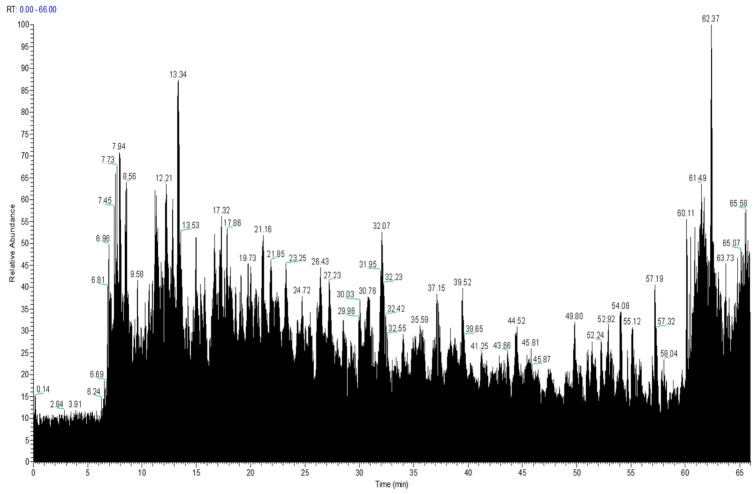
The total ion chromatogram of WP.

After optimizing the WP enzymatic hydrolysate with the best anticoagulant effect using the BBD response surface and Critic-G1 evaluation method, we analyzed the peptide components in the hydrolysate. Through Nano-LC-MS/MS detection, the total ion chromatogram was shown in Figure 3. The homologous proteins primarily involved NADH-ubiquinone oxidoreductase chain, actin, tubulin, GABA receptor, putative voltage-dependent calcium channel, and nicotinic acetylcholine receptor. These proteins participate in various biological processes, including the generation of precursor metabolites and energy, microtubule-based processes, transmembrane transport, regulation of monoatomic ion transmembrane transport, signaling, and nervous system processes.

Using Byonic software 5.9, we identified 309 peptide segments in the WP enzymatic hydrolysate. After removing those with identification scores below 100, 269 peptide segments remained, with an FDR of less than 1% indicating good identification reliability. Furthermore, computer screening was performed to predict biological activity and adverse reactions as well as ADMET properties. Seven peptide segments were selected, as shown in Table 4, and molecular docking was performed on the selected peptides.

### 2.5. Screening and Analysis of Peptides

**Figure 4:**
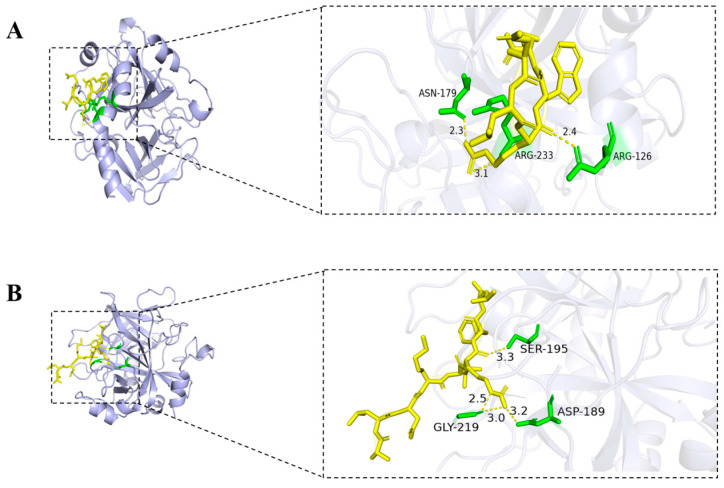

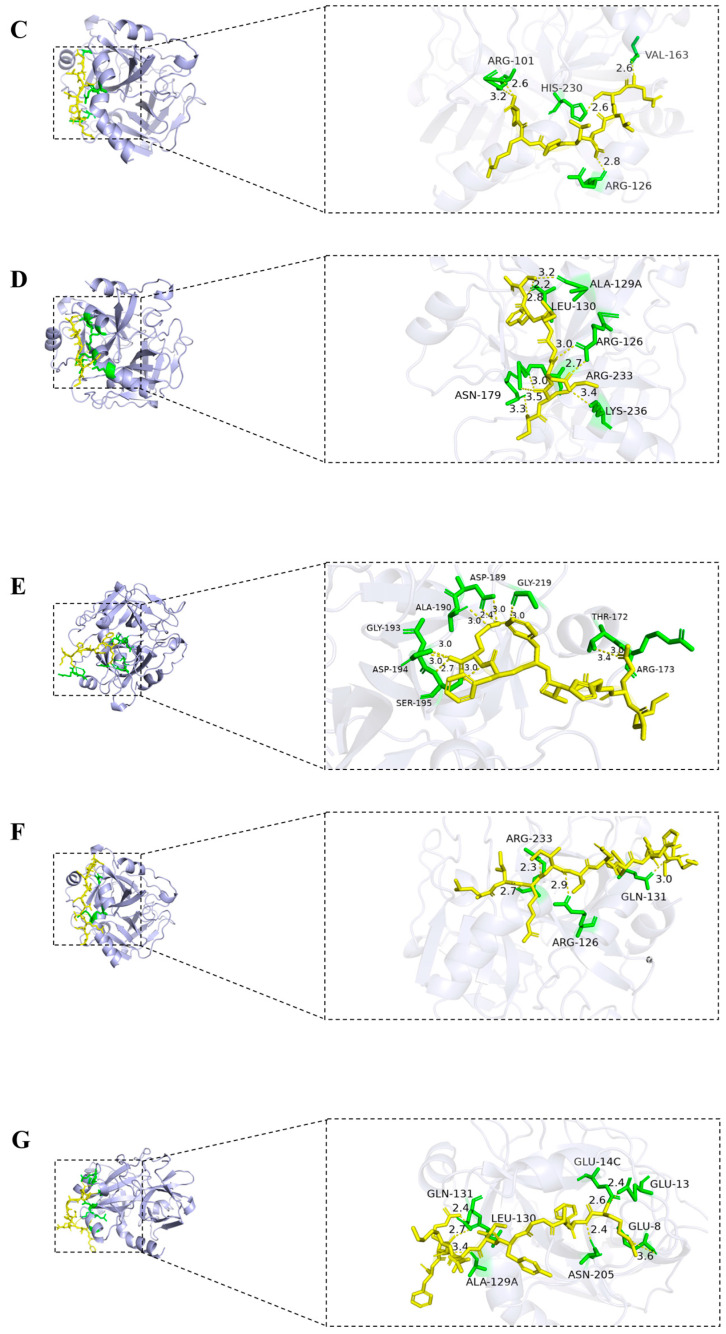
Molecular docking of seven peptide with thrombin; (**A**) DLRWM; (**B**) ALDKIRFL; (**C**) QSLNLPRP; (**D**) CKDGIFT; (**E**) KEHVFFR; (**F**) LIRITDFHLKC; (**G**) RFAGYIEKVRF; hydrogen bonds are shown as yellow dashed lines.

The seven non-toxic peptides selected above were subjected to global docking using the HPEPDOCK server (http://huanglab.phys.hust.edu.cn (accessed on 15 June 2024)). Based on the silico analysis results and HPEPDOCK docking results, one peptide, DLRWM, was preliminarily identified as potentially having anticoagulant activity. Hydrogen bonds are key interactions between thrombin and peptides, helping to improve binding ability and stability. The interaction of the three peptides with thrombin was visualized using PyMOL software 3.1, and the results were shown in Figure 4. DLRWM formed three hydrogen bonds with ARG-233, ASN-179, and ARG-126. ALDKIRFL established four hydrogen bonds involving GLY-219, ASP-189, and SER-195. QSLNLPRP demonstrated five hydrogen bonds with ARG-101, HIS-230, ARG-126, and VAL-163. Notably, CKDGIFT exhibited extensive interactions through nine hydrogen bonds with ASN-179, ARG-233, LYS-236, ARG-126, ALA-129A, and CYS-130. KEHVFFR formed ten hydrogen bonds with ALA-190, ASP-189, GLY-219, GLY-193, ASP-194, SER-195, THR-172, and ARG-173. LIRITDFHLKC generated four hydrogen bonds with ARG-233, ARG-126, and GLN-131, while RFAGYIEKVRF formed seven hydrogen bonds with GLU-8, GLU-13, GLU-14C, ASN-205, ALA-129A, LEU-130, and GLN-131. All seven peptides demonstrated stable hydrogen-bond-mediated binding to thrombin (Table 5).

### 2.6. Peptide Synthesis and Validation

**Figure 5:**
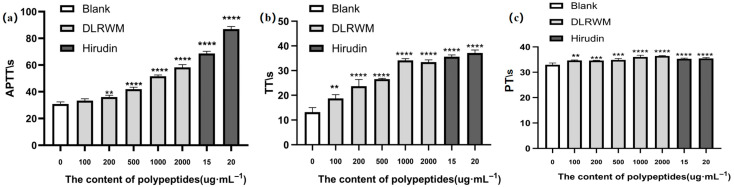
APTT, PT, and TT test results of DLRWM; (**a**) APTT; (**b**) PT; (**c**) TT. (** *p* < 0.01, *** *p* < 0.001, **** *p* < 0.0001).

The selected peptide sequence DLRWM was synthesized by Shanghai Top Biotechnology Co., Ltd. for comprehensive evaluation. The HPLC and mass spectrometry analysis reports are provided in Supplementary Material Figures S1 and S2. As shown in Figure 5, anticoagulant activity assays demonstrated that the synthetic peptide DLRWM significantly prolonged APTT, PT, and TT, indicating its potential anticoagulant capacity.

## 3. Discussion

Coagulation dysregulation plays a pivotal role in thrombotic disorders, with excessive thrombin activity promoting fibrin formation and platelet aggregation. Inhibiting thrombin-mediated pathways and modulating coagulation parameters (APTT, PT, and TT) are critical for antithrombotic therapy [22]. Thrombin, a key serine protease, is generated through both intrinsic and extrinsic pathways, ultimately converting fibrinogen to fibrin [23]. WP has been widely used in TCM for centuries to treat blood stasis and thrombotic disorders, with remarkable clinical efficacy. Its therapeutic effects are attributed to bioactive peptides that target multiple coagulation pathways, offering a natural alternative to synthetic anticoagulants [24]. Unlike single-target drugs such as heparin (which primarily inhibits thrombin) or warfarin (a vitamin K antagonist), leech-derived components exhibit broad-spectrum anticoagulant activity by simultaneously modulating the intrinsic, extrinsic, and common coagulation pathways [25,26]. This study demonstrated that the (WP) peptide DLRWM significantly prolonged APTT, PT, and TT, indicating its capacity to simultaneously target intrinsic, extrinsic, and common coagulation pathways.

The Critic-G1 method successfully integrated four key anticoagulant indicators—activated partial thromboplastin time (APTT, weight 0.1254), prothrombin time (PT, weight 0.2544), thrombin time (TT, weight 0.3726), and peptide content (weight 0.2475)—establishing a comprehensive evaluation system for WP anticoagulant activity. The establishment of this comprehensive anticoagulant evaluation equation represents a significant methodological advancement in the assessment of leech-derived anticoagulants [27,28]. By integrating multiple coagulation parameters through the Critic-G1 weighting system, we have developed a reliable and accurate model for evaluating the anticoagulant activity of WP extracts. The equation provides a quantitative framework that overcomes several limitations of traditional evaluation methods. First, it accounts for the differential contributions of various coagulation pathways, with TT receiving the highest weight (0.3726) due to its direct reflection of thrombin activity and fibrin formation. Second, it incorporates peptide content (weight 0.2475), acknowledging the importance of active component concentration in therapeutic efficacy.

Building on previous studies, we further refined the enzymatic hydrolysis process. Earlier research laid the groundwork for leech peptide extraction and showed that enzymatic hydrolysis enhances anticoagulant activity more effectively than traditional water extraction methods [29]. However, these studies employed fixed hydrolysis conditions without systematic optimization, ultimately resulting in low peptide yields and limited bioactivities. Among these, small-molecule peptides exhibit superior bioavailability and pharmacological activity compared to larger protein fragments [30]. The activity is highly dependent on optimal enzymatic hydrolysis conditions, including temperature, pH, enzyme-to-substrate ratio, and reaction time. The bioactive peptides are influenced by the enzymatic hydrolysis process [31]. Under suboptimal conditions (e.g., excessive temperature or incorrect pH), peptide degradation or misfolding may occur, reducing their therapeutic potential [32]. In this study the Box–Behnken-optimized hydrolysis protocol (50.48 °C, pH 8.06, 7.51 h, 6.78% enzyme-to-substrate ratio) maximizes peptide yield and activity by preserving critical structural motifs. This process ensures the release of low-molecular-weight peptides, which are more readily absorbed and exhibit enhanced anticoagulant effects. The optimized enzymatic hydrolysis process enhances peptide bioavailability. The DLRWM peptide, identified through Nano-LC-MS/MS and bioinformatics screening, shares structural similarities with hirudin’s thrombin-binding domain. Molecular docking revealed that DLRWM forms hydrogen bonds with key residues. It prolongs APTT, PT, and TT. DLRWM’s regulatory effect on thrombin activity underscores its therapeutic potential for abnormal blood coagulation-related disorders. The integration of mass spectrometry, bioinformatics, and molecular docking provides a robust framework for discovering bioactive peptides from traditional medicines, bridging empirical knowledge with modern pharmacology [33].

Our study identified a novel anticoagulant peptide from WP enzymatic hydrolysate while pioneering an innovative strategy for screening bioactive anticoagulant components from protein-based natural medicines. The developed multi-stage approach systematically addresses the following key challenges in peptide drug discovery: (1) developing efficacy-predictive mathematical models through data modeling; (2) optimizing enzymatic hydrolysis processes using model-guided parameters to obtain maximally effective hydrolysates; (3) employing everted gut sac models to identify absorbable peptide fractions; (4) combining peptidomics with molecular docking to characterize and screen bioactive peptides; (5) systematically validating the pharmacological effects of identified peptides.

However, several limitations should be acknowledged. Although in vitro experiments have demonstrated the anticoagulant activity of WP protein hydrolysates, clinical validation is necessary to confirm their therapeutic efficacy and safety in human populations. The complexity of the human circulatory system, along with inter-individual variability in coagulation profiles, may significantly impact the pharmacodynamic outcomes of these peptides. Moreover, the specific bioactive components responsible for the observed anticoagulant effects remain to be fully identified and characterized. While the peptide DLRWM was identified as a promising candidate through molecular docking analysis, the overall pharmacological activity of WP hydrolysates is likely attributed to synergistic interactions among multiple peptide constituents. When critically compared to established anticoagulant peptides such as hirudin and bivalirudin, DLRWM displays several limitations in both structural complexity and pharmacological efficacy, and its structure–function relationships have yet to be thoroughly elucidated. Compared to these well-known anticoagulant peptides, DLRWM presents moderate molecular weight and only partial sequence similarity to the thrombin-binding region of hirudin. Unlike hirudin, DLRWM may exert its anticoagulant effect through a milder, multi-target modulation of intrinsic, extrinsic, and common coagulation pathways, as suggested by its simultaneous prolongation of APTT, PT, and TT. However, the precise molecular mechanism remains unclear. This is due to the absence of defined secondary or tertiary structures in DLRWM, along with limited information on its binding kinetics and in vivo stability. Meanwhile, in this study we synthesized only one peptide (DLRWM) as a representative candidate to validate the feasibility of the screening strategy we established. Validation of other candidate peptides is currently underway and will be included in future work to provide a more comprehensive understanding of their biological activity and mechanisms. It should also be noted that the initial design of this study focused on generating WP protein hydrolysates using trypsin hydrolysis alone, with the aim of enriching potential anticoagulant peptides for further screening. However, this approach inherently overlooked the complex enzymatic environment encountered during gastrointestinal digestion in vivo. Peptides identified through trypsin hydrolysis may represent only transient intermediates that are further degraded by pepsin, chymotrypsin, carboxypeptidases, and other digestive enzymes, which could significantly affect their stability and biological activity in the human body. As a result, the stability, integrity, and functional persistence of these peptides under physiological conditions remain uncertain. There is a real possibility that the bioactive peptides identified in vitro may not survive the digestive process intact, thereby altering or even abolishing their anticoagulant potential when administered orally. Therefore, future studies should incorporate simulated gastrointestinal digestion models, such as the INFOGEST protocol [34], or conduct in vivo assessments to evaluate peptide degradation, absorption, pharmacokinetics, and bioavailability in a more realistic biological context. Additionally, since the stability of peptides cannot be determined, methods to protect them—including traditional capsules/tablets with advanced enteric coatings, microencapsulation systems using biodegradable polymers like alginate or chitosan to control release and reduce enzymatic degradation, and novel formulations with stabilizers such as antioxidants or cyclodextrins—should be prioritized for further investigation.

Future research should aim to isolate, characterize, and standardize these active components to advance drug development and facilitate clinical translation. Meanwhile, intestinal absorption was examined without distinguishing specific segments or assessing concentration-dependent kinetics. More studies should explore region-specific absorption, pharmacokinetics, and bioavailability factors to optimize formulation. The current work focused on in vitro evaluation of synthetic peptides (e.g., DLRWM). In addition, the stability of these peptides during digestion is still unclear and needs further study to confirm if they can reach the intestine in their original form. Further in vivo studies are needed to confirm efficacy, clarify mechanisms, and assess interactions with coagulation factors. The long-term safety of WP-derived peptides also requires evaluation through chronic toxicity studies and clinical trials. Addressing these limitations will enhance understanding of WP’s therapeutic potential and support its clinical application for blood stasis-related disorders.

## 4. Materials and Methods

### 4.1. Materials and Reagents

*Whitmania pigra* Whitman used in this study was collected from anguo medicinal materials market, Hebei Province of China. It was identified by Professor Yang Yaojun of the Department of Traditional Chinese Medicine Identification, School of Traditional Chinese Medicine, Beijing University of Traditional Chinese Medicine. The WP was grinded into a fine powder using a high-speed disintegrator. The powder was passed through a 50-mesh sieve for further use.

An activated partial thromboplastin time assay kit (APTT, STY20201-63-1), prothrombin time assay kit (PT, STY20101-58-4), and thrombin time assay kit (TT, STY20301-41-4) were purchased from Taizhou Zhongqin Shidi Biotechnology Co., Ltd. (Taizhou, China); Rabbit Platelet-Poor Plasma (PPP, 20210775234), trypsin (1:250, 250 U/mg, from porcine pancreas, R21J9D63766) pepsin (1:30,000, from porcine gastric mucosa, M17IS215176), and phosphate-buffered saline (pH = 8.0), BCA Protein Assay Kit (KGP902) were purchased from Shanghai yuanye Bio-Technology Co., Ltd. (Shanghai, China). Acetonitrile (mass spectrometry grade), formic acid (mass spectrometry grade), ammonium bicarbonate (mass spectrometry grade), dithiothreitol (analytical grade), and iodoacetamide (analytical grade) were all purchased from Sigma-Aldrich. Ultrapure water was purified by a Millipore water purification system with a resistivity of 18.2 MΩ·cm. All other reagents and chemicals used were of analytical or biological grade.

Male SPF-grade SD rats (Certificate No. Of SCXK (JING)-2019-0010), weighing 180–220 g, were purchased from SBF (Beijing) Biotechnology Co., Ltd. (Beijing, China). All experimental animals were maintained under controlled environmental conditions in the Beijing University of Chinese Medicine Animal Facility.

### 4.2. The Establishment of a Mathematical Model Based on Anticoagulation Indicators

#### 4.2.1. Preparation of Enzymolysis Samples of WP

The preparation method for WP samples was adapted from the previous method described by Chai et al. with a slight modification [27]. The powder was accurately weighed and added to the PBS solution at a ratio of 1:10 (g:mL). The pH was adjusted to 7.0, and the mixture was placed in a water bath at 50 °C. A precise volume of 10% trypsin was added, and hydrolysis was conducted for 6 h. Subsequently, the hydrolysate was transferred to an 85 °C water bath for 15 min to inactivate the enzyme. After cooling the solution to room temperature, it was centrifuged at 4000 r·min^−1^ for 15 min. The supernatant was collected and freeze-dried.

#### 4.2.2. Everted Gut Sac Preparation

According to previous studies [35], the everted gut sac procedure was performed as follows: An appropriate amount of WP hydrolysate was dissolved in Tyrode’s solution to a final concentration of 50 mg·mL^−1^. Rats were fasted for 12 h with free access to water. After intraperitoneal injection of 20% urethane, the abdominal cavity was opened and the small intestine was quickly excised and rinsed in Tyrode’s solution until clean. An 8 cm segment was harvested from the distal ileum (10–20 cm above the cecum), with mesentery and fat carefully removed. The intestinal segments were gently everted using a glass rod: one end was ligated and the other was connected to a silicone tube to form a sac. Each sac was filled with 2 mL of blank Tyrode’s solution and immersed vertically in a tube containing 15 mL of either 50 mg·mL^−1^ WP hydrolysate or blank Tyrode’s solution, keeping the fluid levels inside and outside balanced. The setup was incubated in a 37 °C water bath with continuous aeration using a gas mixture (95% O_2_, 5% CO_2_) to maintain viability. After 2 h the internal solution was collected and centrifuged at 15,000 rpm for 10 min. The supernatant was used for anticoagulant activity assays.

#### 4.2.3. Determination of Anticoagulation Evaluation Indicators

The peptide content in the WP enzyme hydrolysate samples was determined using a BCA kit. The enzyme hydrolysate samples, treated according to the inverted intestinal sac model, were assessed for anticoagulant activity using a semi-automatic coagulometer. The measurements were conducted in accordance with the instructions for the activated partial thromboplastin time (APTT) assay kit, prothrombin time (PT) assay kit, and thrombin time (TT) assay kit. Clotting times were recorded with blank intestinal fluid serving as the negative control, and the prolongation rates were calculated.

#### 4.2.4. Determination of the Weight Scores for Each Indicator Using the CRITIC-G1 Method

The Critic-G1 hybrid weighting method calculates the weights of process indicators by integrating the Critic and G1 methods. The Critic method standardizes the data of APTT, PT, TT, and WP enzymatic hydrolysis peptides to determine the objective weights. And the G1 method assesses the importance order of the indicators and the relative importance between adjacent ones to derive the subjective weights. These weights are then combined using a multiplicative normalization approach to calculate the combined weight coefficients, as shown in Equation (3). Meanwhile, to evaluate the rationality of the experimental results obtained by the Critic-G1 hybrid weighting method, this study performed a correlation analysis using factor analysis in SPSS 22.0 software. The analysis focused on the comprehensive scores derived from the three weighting methods (Critic, G1, and Critic-G1) as well as the values (combined weights) of Critic and G1. Correlation analysis revealed that the comprehensive scoring results obtained from the three methods (Critic, G1, and Critic-G1) were consistent, leading to the selection of the Critic-G1 method for calculating the composite scores of the indicators. In this context, ${\bar{w}}_{j}$, ${\hat{w}}_{j}$, and wj denote the subjective weight, objective weight, and combined weight, which is depicted as follows:

(3) $$w_{j}=\frac{{\bar{w}}_{j}{\hat{w}}_{j}}{\sum_{j=1}^{m}{\bar{w}}_{j}{\hat{w}}_{j}}$$

### 4.3. Enzymatic Extraction of Optimal Anticoagulant Peptides Based on CRITIC-G1 Model

#### 4.3.1. Single-Factor Investigation

Before applying the BBD methodology, it is first necessary to choose an experimental design that will define which experiments should be carried out in the experimental region being studied [36]. This experiment involves a single-factor study to determine the factors and their levels of Box–Behnken. The factors selected for the experiment include the type of protease (trypsin, pepsin, or a mixture of both), temperature (45 °C, 50 °C, 55 °C, 60 °C, 65 °C), enzyme-to-substrate ratio (1.25%, 2.5%, 5%, 7.5%, 10%), enzyme digestion time (2 h, 4 h, 6 h, 8 h, 10 h), and enzyme digestion pH (7.6, 7.8, 8.0, 8.2, 8.4). Based on the results of the single-factor experiments, which include measurements of APTT, PT, TT, and WP enzyme hydrolyzed peptide content, a comprehensive score will be calculated using the Critic-G1 weighting method. The optimal extraction factors and their corresponding levels will then be selected. When assessing the impact of a single variable on the content, all other variables are kept constant. Therefore, only one variable was altered at a time to ascertain the optimal conditions.

#### 4.3.2. Box–Behnken Design (BBD) for Optimization of Variables

To optimize the enzymatic hydrolysis process, a Box–Behnken design (BBD) was employed based on preliminary single-factor experiments to determine the levels, central points, and step sizes of the experimental factors, as shown in Table 6. The selected factors included the temperature (A), enzyme-to-substrate ratio (B), enzymatic hydrolysis time (C), and pH value of enzymatic hydrolysis (D). The response variables comprised the prolongation rates of activated partial thromboplastin time (APTT) (y_1_), prothrombin time (PT) (y_2_), thrombin time (TT) (y_3_), and the peptide content in WP hydrolysate (y_4_), which were integrated into a comprehensive score (Y). A four-factor, three-level Box–Behnken design was utilized to investigate the effects of these variables on the enzymatic hydrolysis of WP. Using Design-Expert 8.0.6 software, runs were generated and evaluated. The data were analyzed through second-order multiple regression fitting, including regression analysis and significance testing, to identify the optimal conditions for maximizing anticoagulant activity and peptide yield.

#### 4.3.3. Method Validation

Based on the optimal conditions determined through Design-Expert 8.0.6 and in conjunction with practical operations, three parallel validation experiments were conducted using the predicted optimal enzymatic hydrolysis parameters for WP. The average comprehensive score and the relative standard deviation (RSD) were calculated from these trials. The results were found to closely match the model-predicted values, demonstrating that the enzymatic hydrolysis process exhibits high accuracy and stability.

### 4.4. Nano-LC-MS/MS Analysis

Nano-LC-MS/MS analysis was performed using an Easy-nLC 1200 system (Thermo Fisher Scientific, Waltham, MA, USA) coupled with a Q Exactive™ Hybrid Quadrupole-Orbitrap™ Mass Spectrometer (Thermo Fisher Scientific, Waltham, MA, USA). LC separation was carried out using a Reprosil-Pur 120 C18-AQ column (100 μm × 180 mm, 3 μm) with a flow rate of 600 nL/min. Mobile phase A consisted of 0.1% formic acid in water, and mobile phase B was 0.1% formic acid in 80% acetonitrile. The gradient elution program was as follows: 0–2 min, 4–8% B; 2–45 min, 8–28% B; 45–55 min, 28–40% B; 55–56 min, 40–95% B; and 56–66 min, 95% B. The injection volume was 2 μL.

The mass spectrometry conditions were set as follows: electrospray ionization (ESI) in positive ion mode; data acquisition mode was Full MS-ddMS2 (data-dependent acquisition, DDA); capillary voltage, 3.5 kV; ion transfer tube temperature, 350 °C; Orbitrap resolution, 70,000 for MS1 and 17,500 for MS2; scan range, 300–1800 *m*/*z*; dynamic exclusion, 20 s; collision type, higher-energy collisional dissociation (HCD); normalized collision energy (NCE), 28%. The acquired raw data were processed using Byonic software 5.9 (Protein Metrics, San Carlos, CA, USA) for database searching. The database used was WP, and the search parameters were set as follows: fixed modification, carbamidomethylation (C); variable modifications, oxidation (M) and acetylation (peptide N-terminus); enzyme, non-specific; peptide mass tolerance, 20 ppm; fragment mass tolerance, 0.02 Da. The search results were filtered to achieve a false discovery rate (FDR) of less than 1% for both peptide and protein identifications.

### 4.5. Research on Computer Screening and Bioinformatics for Antithrombotic Peptides

Peptide segments were identified by comparing with the existing Uniprot database, which includes leech species such as Hirudo medicinalis (Medicinal leech), Hirudo verbana, and Hirudo nipponia. To evaluate the toxicity, solubility, and allergenicity of these high-scoring peptides, they were submitted to ToxinPred (https://webs.iiitd.edu.in/raghava/toxinpred/ (accessed on 20 July 2024)), Innovagen (http://www.innovagen.com/proteomics-tools/ (accessed on 20 July 2024)), and AllergenFP (https://ddg-pharmfac.net/AllergenFP (accessed on 20 July 2024)), respectively.

Peptides that were non-toxic, soluble, and exhibited no potential allergenicity were selected. The selected peptides were converted into Simplified Molecular Input Line Entry System (SMILES) format using the “SMILES” tool from BIOPEP-UWM (https://biochemia.uwm.edu.pl/biopep-uwm/ (accessed on 20 July 2024)). These SMILES-formatted peptides were subsequently submitted to the ADMET Lab (https://admetmesh.scbdd.com/ (accessed on 20 July 2024)) website to filter out those with low blood–brain barrier (BBB) penetration.

### 4.6. Molecular Docking

Thrombin was selected as the target of coagulation activity, and its specific structural information (PDB: 2BVR) was extracted from the PDB protein structure database (RCSBPDB, https://www.rcsb.org/ (accessed on 20 July 2024)). PyMOL software was used to remove water, hydrogenate, and delete unnecessary 4CP ligands in the crystal structure. Peptides with identification scores greater than 100, non-toxic, and molecular characteristics consistent with antithrombotic peptides were selected as ligands for molecular docking experiments with thrombin. Global molecular docking [37] was performed by HPEPDOCK server (http://huanglab.phys.hust.edu.cn (accessed on 18 July 2024)), and hirudin variant (GDFEEIPEEYLQ) was used as a positive control. Finally, the peptides were screened according to the docking scores, and the intermolecular interaction analysis was performed using PyMOL software to find the optimal conformation.

### 4.7. Synthesis of Peptide and Validation of Anticoagulant Activity

The peptide segment DLRWM, which has higher activity and a higher thrombin-binding score, was selected for synthesis verification. Peptide was synthesized using solid-phase synthesis using the Fmoc method, conducted by Shanghai Top Biotechnology Co., Ltd. (Shanghai, China), attaining a purity surpassing 95%. The purity and molecular weight of the synthesized peptides were determined using HPLC and liquid chromatography–mass spectrometry (LC-MS), respectively. Using hirudin as the positive control, the anticoagulant activity (APTT, PT, and TT) of synthetic peptides at varying concentrations was assessed following the method in Section 4.2.2.

### 4.8. Statistical Analysis

All data were expressed as mean ± SD. Statistical analyses were performed using one-way ANOVAs and *t*-tests with GraphPad Prism 8.0.

## 5. Conclusions

In this study, a novel anticoagulant evaluation model was established for the first time by applying the Critic-G1 method to determine the weight coefficients of four key indicators: activated partial thromboplastin time (APTT, 0.3726), prothrombin time (PT, 0.2544), thrombin time (TT, 0.2475), and WP peptide content (0.1254). Using this comprehensive model as the benchmark, the enzymatic hydrolysis process of WP was optimized through single-factor experiments combined with Box–Behnken response surface methodology. The optimal conditions were identified as follows: trypsin hydrolysis at 50.48 °C, enzyme-to-substrate ratio of 6.78%, hydrolysis time of 7.51 h, and pH 8.06. Subsequently, the everted gut sac model was employed to screen peptides capable of crossing the intestinal barrier from the optimal anticoagulant hydrolysate. Nano-LC-MS/MS analysis of the hydrolysate revealed a potent anticoagulant peptide, DLRWM, which significantly prolonged APTT, PT, and TT, respectively. This study elucidates bioactive peptides of WP’s anticoagulant activity. The integration of mathematical modeling, enzymatic optimization, and advanced peptidomics techniques offers a new paradigm for researching bioactive components in animal-based traditional medicines and supports their clinical application.

## Figures and Tables

**Table 1:** APTT, PT, and TT determination results of WP enzymatic hydrolysis samples ($\bar{x}\;\pm \;s$, *n* = 3).

Biological Repetition	APTT Prolongation Rate (%)	PT Prolongation Rate (%)	TT Prolongation Rate (%)	Peptide Content (mg·g^−1^)
1	31.68 ± 1.81	18.05 ± 2.28	102.00 ± 2.03	210.6085 ± 1.46
2	33.68 ± 2.81	18.15 ± 2.44	104.62 ± 2.82	210.0794 ± 0.32
3	33.26 ± 0.67	16.73 ± 1.50	120.17 ± 0.89	215.3704 ± 0.82
4	35.98 ± 1.51	16.50 ± 2.58	107.47 ± 2.94	214.9735 ± 1.63
5	33.83 ± 2.18	18.08 ± 0.74	106.31 ± 1.54	208.3598 ± 0.49
6	29.86 ± 1.06	16.30 ± 2.46	107.62 ± 1.73	211.4021 ± 0.75

**Table 2:** Weight values of evaluation indicators for PT, APTT, TT, and WP enzyme hydrolysis peptide content.

Evaluation Indicators	*W*_1_	*W*_2_	*W*
APTT prolongation rate	0.1399	0.2217	0.1254
PT prolongation rate	0.2039	0.3087	0.2544
TT prolongation rate	0.4001	0.2304	0.3726
Peptide content	0.2561	0.2391	0.2475

**Table 3:** Comparison of comprehensive scores for three weighting methods.

No.	CRITIC	G1	CRITIC-G1
1	91.60	93.16	92.17
2	93.30	95.00	93.76
3	97.35	95.90	97.05
4	93.87	94.70	93.69
5	93.64	95.11	94.04
6	90.89	90.23	90.92

**Table 4:** Selected anticoagulant peptide segments of WP.

No	Peptide	Bioactivity	Toxicity	Water Solubility	Blood–Brain Barrier	Allergenicity
1	DLRWM	0.897687	Non-Toxin	Good water solubility	Low	Non-allergenic
2	ALDKIRFL	0.775583	Non-Toxin	Good water solubility	Low	Non-allergenic
3	QSLNLPRP	0.699158	Non-Toxin	Good water solubility	Low	Non-allergenic
4	CKDGIFT	0.620344	Non-Toxin	Good water solubil	Low	Non-allergenic
5	KEHVFFR	0.573015	Non-Toxin	Good water solubility	Low	Non-allergenic
6	LIRITDFHLKC	0.536102	Non-Toxin	Good water ity	Low	Non-allergenic
7	RFAGYIEKVRF	0.526837	Non-Toxin	Good waterlity	Low	Non-allergenic

**Table 5:** Peptide and thrombin docking fraction.

No	Polypeptide Sequence	HPEPDOCK Docking Score
Hirudin control	GDFEEIPEEYLQ	−143.292
1	DLRWM	−195.992
2	ALDKIRFL	−183.342
3	QSLNLPRP	−184.073
4	CKDGIFT	−162.272
5	KEHVFFR	−193.025
6	LIRITDFHLKC	−196.705
7	RFAGYIEKVRF	−194.394

**Table 6:** Factors and levels of Box–Behnken response surface methodology for WP enzymatic hydrolysis process.

Level		Factor		
	Temperature/°C (A)	Enzyme-to-Substrate Ratio/% (B)	Time/h (C)	PH (D)
−1	45	5	4	7.8
0	50	7.5	6	8.0
1	55	10	8	8.2

## Data Availability

The data are confidential. Further inquiries can be directed to the corresponding author.

## Supplementary Materials

The following supporting information can be downloaded at: https://www.mdpi.com/article/10.3390/molecules30153184/s1, Figures S1 and S2: HPLC and MS Analysis of DLRWM; Tables S1 and S2: Box Behnken Response Surface Methodology Results and Analysis of variance results of fitting models of Leech Enzymatic Hydrolysis Process.

## Abbreviations

The following abbreviations are used in this manuscript:

WP	*Whitmania pigra* Whitman
TCM	Traditional Chinese Medicine
RSM	Response Surface Methodology
BBD	Box–Behnken Design
APTT	Activated Partial Thromboplastin Time
TT	Thrombin Time
PT	Prothrombin Time
