# Supplementary material for: A Novel Integrated Strategy for Discovering Absorbable Anticoagulant Bioactive Peptides: A Case Study on Leech Protein Hydrolysates
Source: Molecules. 2025 Jul 30;30(15):3184. doi: 10.3390/molecules30153184 (PMC12348763; doi:10.3390/molecules30153184)

Table S1 Box Behnken Response Surface Methodology and Results of Leech Enzymatic Hydrolysis Process

Run	A/℃	B/%	C/h	D	y1/%	y2/%	y3/%	y4/（$mg\cdot g^{-1}$）	Y
1	50	7.5	6	8	61.64±0.14	30.88±0.47	99.20±2.82	244.54±0.73	65.64
2	45	10	6	8	19.90±0.51	5.43±2.18	159.80±2.60	203.23±1.57	55.85
3	55	5	6	8	69.20±2.73	5.19±0.81	52.93±0.14	299.81±2.22	41.80
4	55	7.5	6	7.8	69.85±2.93	6.98±0.23	64.75±0.29	278.59±2.98	44.48
5	50	5	6	7.8	5.86±0.36	7.64±0.13	6.16±0.38	218.76±0.60	21.11
6	55	7.5	6	8.2	12.44±0.35	21.70±0.23	17.27±0.74	320.04±0.60	40.29
7	50	10	6	8.2	11.24±1.39	1.32±0.13	89.09±0.43	243.26±1.76	38.28
8	50	10	8	8	37.13±0	17.24±0.56	116.06±0.86	188.85±1.21	54.40
9	50	7.5	6	8	69.26±2.83	20.92±2.99	125.76±0.94	300.38±1.85	69.43
10	55	7.5	4	8	70.18±1.59	15.03±2.63	30.61±0.25	211.92±1.74	37.73
11	50	5	4	8	65.54±2.49	17.44±0.21	32.02±0.38	224.46±0.40	40.02
12	50	7.5	8	8.2	111.15±1.39	26.04±0.65	68.39±0.68	274.03±1.41	61.98
13	50	7.5	8	7.8	73.23±1.87	13.46±1.04	67.37±1.22	305.09±2.63	51.48
14	50	7.5	6	8	88.64±0.09	20.92±2.95	129.08±2.82	267.91±0.60	70.05
15	50	7.5	6	8	115.45±1.86	22.34±0.68	125.90±1.69	253.52±3.22	72.01
16	50	7.5	4	7.8	126.66±1.09	23.30±0.15	32.64±0.54	300.81±0.60	55.03
17	45	7.5	4	8	52.68±0.07	37.74±0.28	81.52±0.99	342.69±1.05	71.51
18	50	5	6	8.2	110.38±2.64	17.96±1.78	62.32±2.16	388.28±1.41	62.32
19	50	7.5	4	8.2	89.74±2.19	12.08±2.93	36.36±0.89	325.46±1.41	46.25
20	50	10	6	7.8	37.31±2.90	10.02±0	148.48±2.14	253.66±1.07	61.24
21	50	7.5	6	8	64.43±0.05	14.65±0.10	147.01±2.25	334.72±0.40	71.87
22	50	10	4	8	32.11±2.83	7.39±0.46	136.97±1.71	257.36±1.65	56.50
23	45	5	6	8	109.89±2.39	16.73±0.29	40.10±0.14	307.08±2.82	51.08
24	55	7.5	8	8	43.10±0.46	31.79±0.71	107.98±2.86	287.85±0.73	69.23
25	45	7.5	6	8.2	48.75±2.60	32.36±0	39.80±1.00	360.64±2.79	58.91
26	45	7.5	8	8	57.67±0.43	31.54±0.72	40.71±0.62	363.92±0.20	59.66

27	50	5	8	8	70.98±0.50	25.50±0.74	58.99±0.76	335.57±1.72	59.37
28	45	7.5	6	7.8	24.20±2.13	16.38±3.57	51.62±0.29	343.12±1.81	47.35
29	55	10	6	8	12.40±2.82	5.80±1.67	152.02±3.71	179.44±1.05	52.02

Table S2 Analysis of variance results of fitting models

Source	Sum of squares	df	Mean square	F-value	P-value
Model	4303.92	14	307.42	37.47	< 0.0001
A	288.23	1	288.23	35.13	< 0.0001
B	151.25	1	151.25	18.43	0.0007
C	200.67	1	200.67	24.46	0.0002
D	62.24	1	62.24	7.59	0.0155
AB	7.45	1	7.45	0.91	0.3569
AC	469.68	1	469.68	57.24	< 0.0001
AD	62.03	1	62.03	7.56	0.0157
BC	115.04	1	115.04	14.02	0.0022
BD	1029.12	1	1029.12	125.42	< 0.0001
CD	92.95	1	92.95	11.33	0.0046
A2	388.66	1	388.66	47.37	< 0.0001
B2	970.12	1	970.12	118.23	< 0.0001
C2	83.35	1	83.35	10.16	0.0066
D2	1077.57	1	1077.57	131.33	< 0.0001
Residual	114.87	14	8.21		
Lack of Fit	88.19	10	8.82	1.32	0.4236
Pure Error	26.68	4	6.67		
Cor Total	4418.79	28			

Figure S1 HPLC Analysis of DLRWM

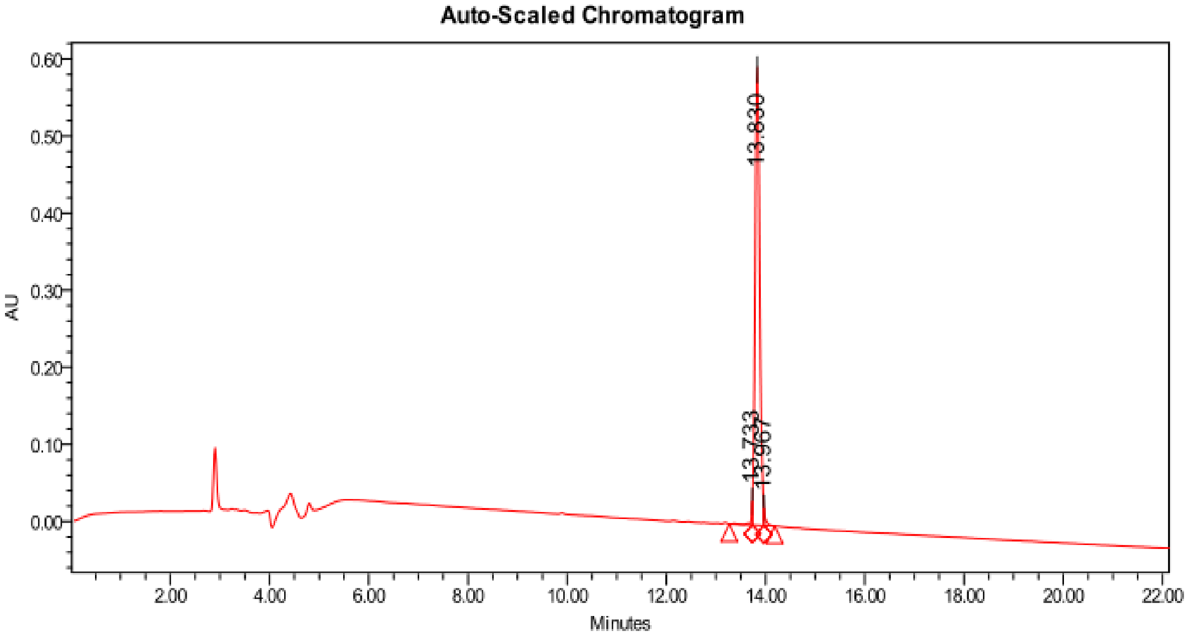

Peak Results

	RT	Area	Height	% Area
1	13.733	45434	30541	1.12
2	13.830	3976967	593660	97.65
3	13.967	50432	22321	1.24

Figure S2 MS Analysis of DLRWM

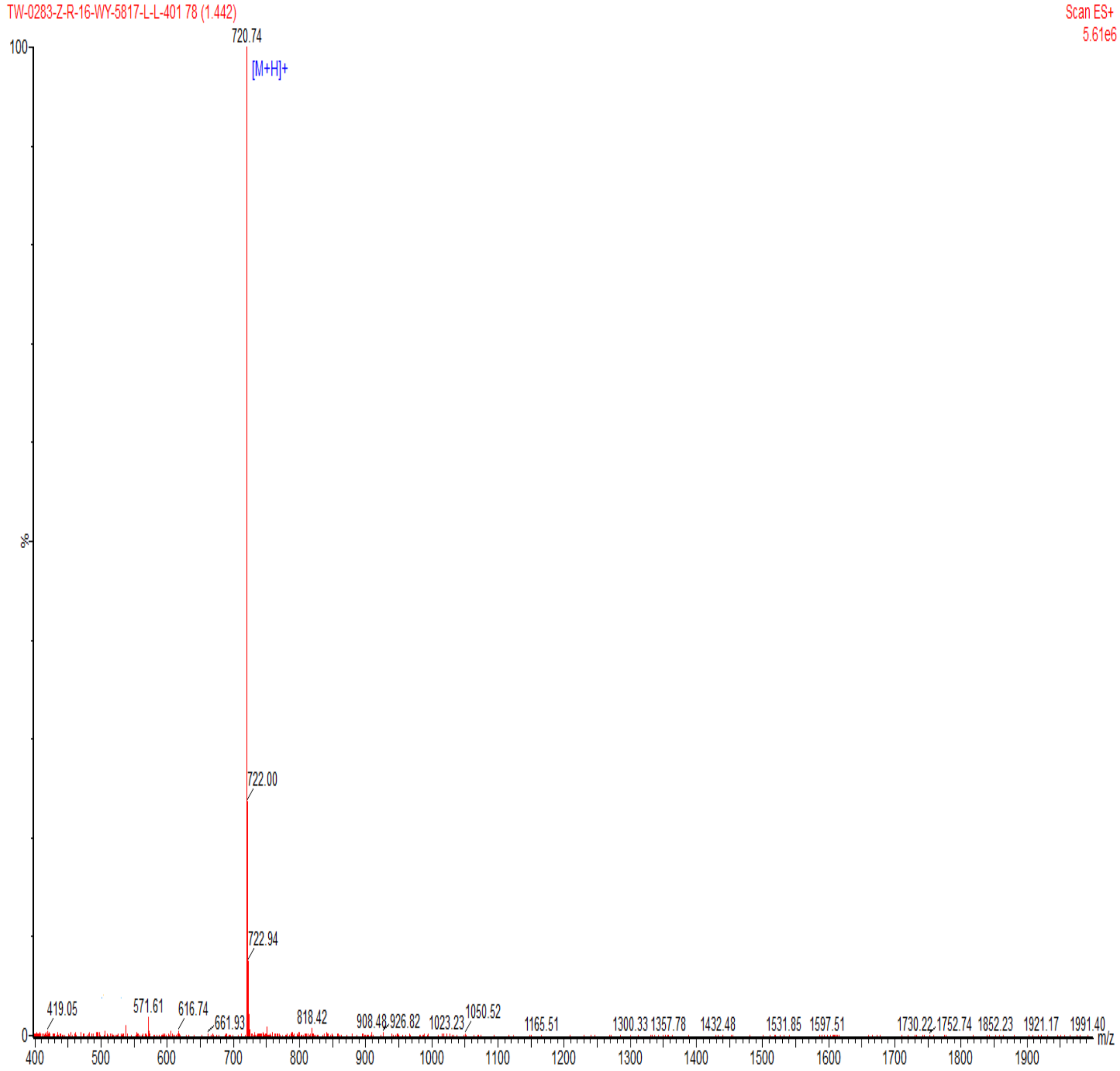

Supplement: Supplementary file 1 [file molecules-30-03184-s001.zip › molecules-3724046-supplementary.pdf]
